# HOXA10 enhances cell proliferation and suppresses apoptosis in esophageal cancer via activating p38/ERK signaling pathway

**DOI:** 10.1515/med-2022-0558

**Published:** 2022-11-03

**Authors:** Lifeng Jiang, Qixian Yang

**Affiliations:** Department of Gastroenterology, The Affiliated Changzhou No. 2 People’s Hospital of Nanjing Medical University, Changzhou, Jiangsu, 213003, China; Clinical Laboratory of Diagnostics and Gastroenterology, The Affiliated Changzhou No. 2 People’s Hospital of Nanjing Medical University, No. 68 Gehuzhonglu Road, Wujin District, Changzhou, Jiangsu, 213003, China

**Keywords:** HOXA10, esophageal cancer, proliferation, apoptosis, ERK signaling pathway, p38 signaling pathway

## Abstract

Esophageal cancer (EC) is an extremely aggressive malignant tumor. Homeobox A10 (HOXA10) is highly expressed and plays an important role in a variety of tumors. However, the function of HOXA10 in EC remains unclear. In this study, HOXA10 was observed to highly express in EC tissues and cells. Interestingly, the CCK-8 assay, flow cytometry, and colony formation assay confirmed that overexpression of HOXA10 promoted proliferation and suppressed cell apoptosis in EC cells. More importantly, the western blot assay indicated that the phosphorylation levels of ERK and p38 were elevated in EC cells overexpressed HOXA10, indicating that overexpression of HOXA10 activated p38/ERK signaling pathway in EC cells. These findings concluded that HOXA10 aggravated EC progression via activating p38/ERK signaling pathway, providing a potential therapeutic target for EC.

## 1 Introduction

Esophageal cancer (EC) is a common tumor of the digestive tract, which is the most aggressive of all cancerous tumors of the gastrointestinal tract. Owing to the inconspicuous early symptoms of EC and the difficulty of diagnosed, the mortality rate of EC is very high, and the 5-year survival rate is very short [1]. Although the main therapeutic strategies of EC are chemoradiotherapy and surgical resection, the recurrence rate and mortality rate after resection remain still very high [2]. With the gradual improvement of medical level, personalized treatment or precision medicine and treatment methods based on some biomarkers are further developed [3,4]. Therefore, it is very important to find new reliable therapeutic targets and biomarkers in EC and investigate the cellular changes that cause metastasis and diffusion for the diagnosis and treatment of EC.

Homeobox A10 (HOXA10) is a member of the HOX gene family, which can be divided into four subtypes: A, B, C, and D, whose expression in the embryonic stage is regulated by time and space. HOXA10 is highly expressed in a variety of tumors, which has been confirmed to participate in the tumorigenesis. In bladder cancer, HOXA10 can upregulate MMP-3 and promote the invasion and proliferation of cancer cells [5]. Moreover, HOXA10 can promote proliferation and inhibit apoptosis of gastric cancer cells [6]. Besides, HOXA10 induces apoptosis of hepatocellular carcinoma cells by targeting HDAC1 [7]. Overexpression of HOXA10 is closely related to the poor prognosis of acute myeloid leukemia [8]. A recent study has reported that the expression of HOXA10 is increased in EC, whereas the specific mechanism has not been explored [9].

The representative hallmark of cancer is unscheduled proliferation, and p38 and ERK signaling pathways participated in the regulation of cell survival and apoptosis, which have profound effects on tumorigenesis. It has been reported that HOXA10 can activate ERK and p38 signaling pathways, thereby regulating cell proliferation and apoptosis [10]. Abnormal activation of ERK and p38 signaling pathway is related to the occurrence and development of EC; even more, activation of P38 and ERK pathway can promote the progression of EC [11,12,13]. However, whether HOXA10 can participate in the progression of EC by regulating p38 and ERK pathways needs further study. This study mainly explored the specific role and mechanism of HOXA10 in EC.

## 2 Methods and materials

### 2.1 HOXA10 profile data collection

The data of HOXA10 expression in 11 normal samples and 184 esophageal carcinoma (ESCA) samples were downloaded from TCGA database (https://tcga-data.nci.nih.gov/tcga/). Besides, the expression of HOXA10 in ESCA and normal subjects was downloaded from GEPIA (gepia.cancer-pku.cn) and UCSC Xena database (xena.ucsc.edu), respectively.

### 2.2 Cell culture and transfection

The normal human esophageal epithelial cell line (HEEC; catalog: ZQ0989) was purchased from Zhongqiaoxinzhou (China), the EC9706 (catalog: 3,567) cell line was obtained from TOKU-E (USA), and the human Eca-109 (catalog: 1101HUM-PUMC000246), TE-1 (catalog: 1101HUM-PUMC000986), and KYSE150 (catalog: 3101HUMTCHu236) cell lines were purchased from Cell Culture Center of the Chinese Academy of Medical Sciences. All the cells above were cultured in RPMI-1640 (Gibco, USA) complete culture medium containing 10% fetal bovine serum (Gibco) at 37°C in 5% CO_2_.

The TE-1 and Eca-109 cells were transfected with 2 μg of pcDNA3.1-HOXA10 or empty pcDNA3.1 by using Lipofectamine 2000 (Invitrogen, USA). The siRNAHOXA10 and its corresponding control siNC were synthesized by Ribobio (Guangzhou, China). For HOXA10 knockdown, the Eca-109 and TE-1 cells were treated with siHOXA10 or siNC, respectively.

### 2.3 Cell viability assay

After transfection, the TE-1 and Eca-109 cells were seeded into 96-well plates and incubated for 24, 48, and 72 h. Thereafter, each well was added with 10 µL CCK-8 solution (Beyotime, China) for 2 h incubation at 37°C. Finally, the absorbance of each well at 450 nm was measured by a microplate reader.

### 2.4 Colony formation assay

After transfection, a total of approximately 500 cells per well were seeded in six-well plates for 14 days, fixed with methanol (Beyotime, China), and then stained with 0.1% crystal violet (Sigma, China). The colonies containing at least 50 cells were counted.

### 2.5 Cell apoptosis assay

After transfection, the Eca-109 and TE-1 cells were collected. Subsequently, the cell apoptosis was determined by Annexin V-fluorescein isothiocyanate (FITC) cell apoptosis kit (Beyotime, China). A total of 5  ×  10^5^ cells were resuspended in binding buffer and then stained with Annexin V-FITC solution for 20 min in the dark. The cell apoptosis was captured under a flow cytometry (BD FACSCalibur, USA).

### 2.6 Quantitative RT-PCR assay

The total RNA from cells was extracted by RNAsimple Kits (TIANGEN, China). The total RNA was then reverse-transcribed into cDNA by TIANscript RT Kit (TIANGEN, China). Then, the *HOXA10* mRNA level was detected by SYBR Green Reagents (Bio-Rad, USA). The relative HOXA10 expression was calculated with 2^−ΔΔCt^ method: *HOXA10*-F: 5′-ACAAGCACACCACAATTCTCC-3′ and *HOXA10*-R: 5′-ATCCAAACAATGTCTCCCTTCTC-3′; *GAPDH*-F: 5′-ACCACAGTCCATGCCATCAC-3′ and *GAPDH*-R: 5′-TCCACCACCCTGTTGCTGTA-3′.

### 2.7 Western blot assay

The cells or tissues were lysed in RIPA buffer, separated by sodium dodecyl sulfate polyacrylamide gel electrophoresis, and then transferred onto polyvinylidene fluoride membranes. Subsequently, the membranes were incubated with 5% skim milk and then blocked with the specified primary antibodies overnight at 4°C, including HOXA10 (1/800, Abcam, UK), Bax (1/800, Abcam), Bcl-2 (1/800, Abcam), p-ERK (1/800, Abcam), cleaved caspase-9 (1/800, Abcam), cleaved PARP (1/800, Abcam), ERK (1/800, Abcam), p-p38 (1/800, Abcam), cleaved caspase-3 (1/800, Abcam), p38 (1/800, Abcam), PARP (1/800, Abcam), and GAPDH (1/800, Abcam). Next, the membranes were then blocked with appropriate secondary antibodies (1/5,000; Beyotime, China) at room temperature for 2 h. Protein bands were visualized using BeyoECLStar (Beyotime). The quantification of western blots was measured by the intensity of bands (ImageJ, USA).

### 2.8 Statistical analysis

The results were summarized as the mean ± standard deviation. The comparison was performed by *t*-tests or one-way analysis of variance though SPSS 20.0 software (USA). The *p* < 0.05 was considered as statistical significance.

## 3 Results

### 3.1 HOXA10 was highly expressed in EC tissues and cells

**Figure 1:**
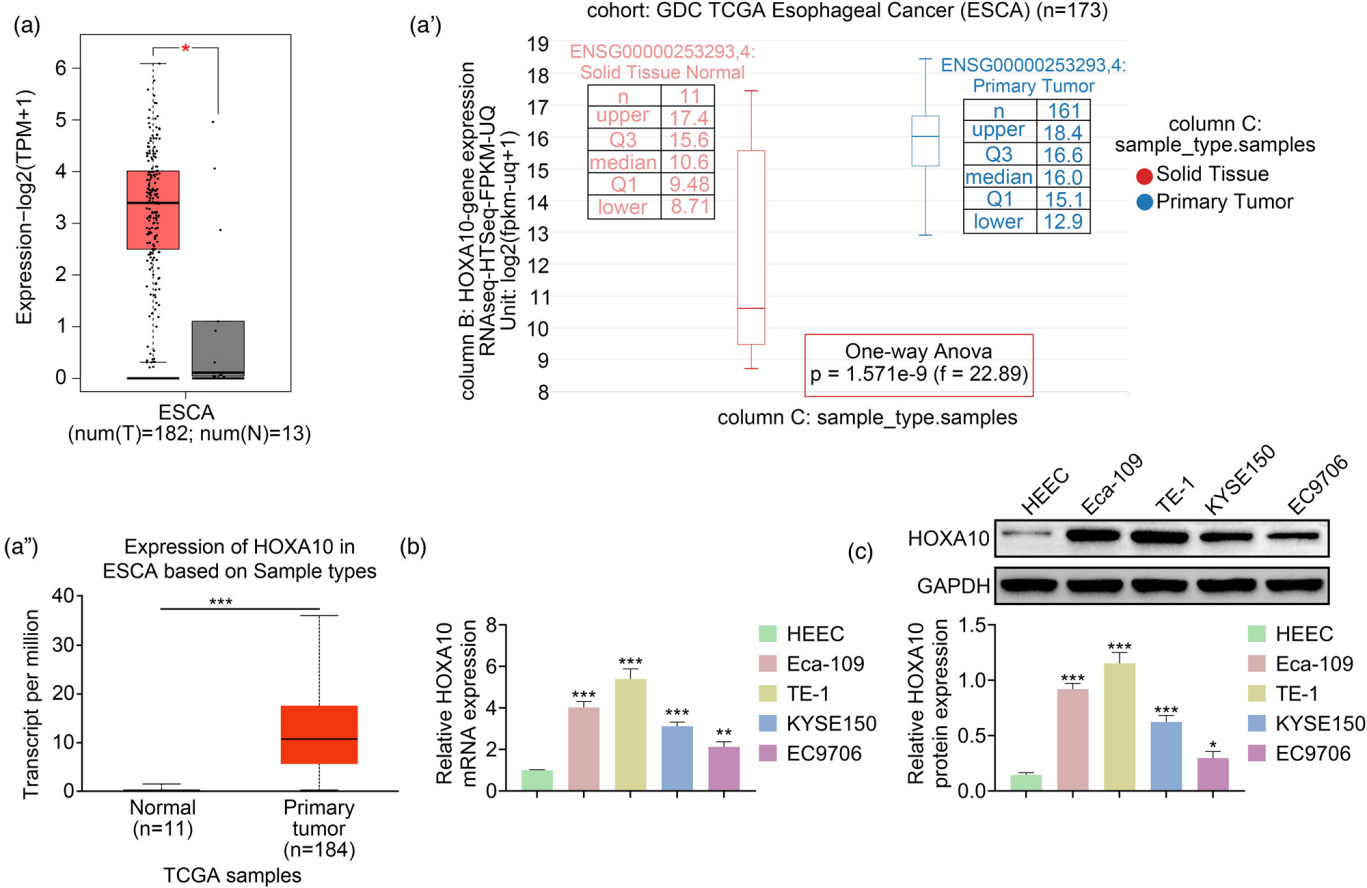
HOXA10 was highly expressed in EC tissues and cells. (a) The HOXA10 expression was elevated in primary tumor tissues, which was analyzed by TCGA (a), GEPIA (a′), and UCSC Xena (a″) databases. (b) The *HOXA10* mRNA level was increased in EC cell lines containing Eca-109, EC9706, TE-1, and KYSE150. (c) The HOXA10 protein level was promoted in EC cell lines. Each experiment repeated three times. ^*^ *p*  < 0 .05, ^**^ *p*  <  0.01, ^***^ *p*  <  0.001.

To explore the HOXA10 expression in EC, TCGA data indicated that HOXA10 was observed to elevate in tumor tissues (Figure 1a, a′ and a″). In addition, real-time PCR results demonstrated that *HOXA10* mRNA level was higher in EC cell lines (Eca-109, EC9706, TE-1, and KYSE150) than in HEEC cells (Figure 1b). Consistently, the HOXA10 protein level was significantly higher in EC cell lines than in HEEC cells (Figure 1c). Therefore, these findings proved that HOXA10 expression was enhanced in EC tissues and cells.

### 3.2 Overexpression of HOXA10 promoted proliferation in EC cells

**Figure 2:**
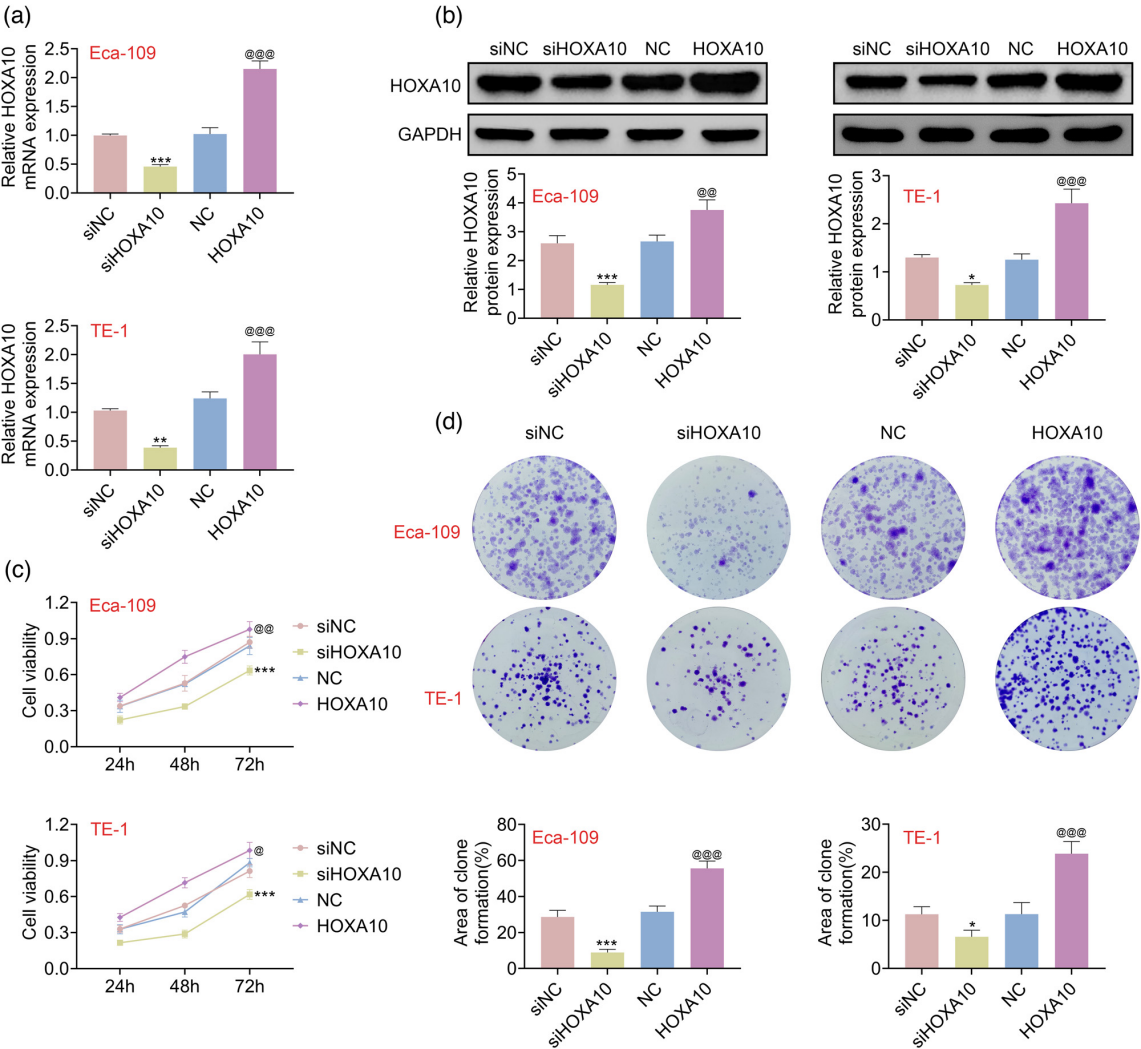
Overexpression of HOXA10 enhanced proliferation in EC cells. (a) Real-time PCR showed *HOXA10* mRNA level in Eca-109 and TE-1 cells. (b) Western blot showed HOXA10 protein level in Eca-109 and TE-1 cells. (c) CCK-8 assay revealed that the elevation of cell viability was caused by HOXA10 overexpression and inhibition of cell viability was induced by HOXA10 knockdown. (d) Clone formation assay determined that HOXA10 overexpression increased the colony number and HOXA10 knockdown suppressed the colony number. Each experiment repeated three times. ^**^ *p*  <  0.01, ^***^ *p*  < 0 .001 vs NC. ^@@^ *p* < 0.01, ^@@@^ *p* < 0.001 vs siNC.

**Figure A1:**
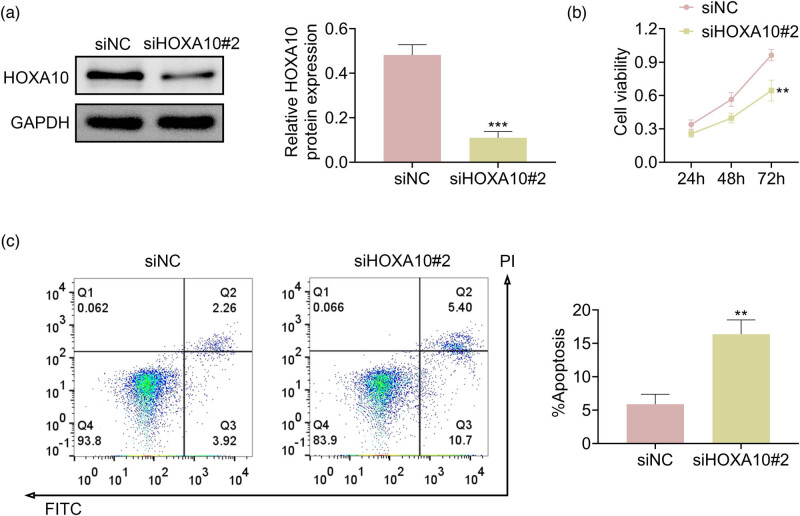
Knockdown of HOXA10 reduced cell proliferation and enhanced apoptosis in EC cells. (a) The western blot showed HOXA10 expression in Eca-109 cells. (b) The cell viability in Eca-109 cells was determined by CCK-8 assay. (c) The cell apoptosis in Eca-109 cells was detected by Annexin V-FITC assay.

Among the EC cell lines, HOXA10 expression was highest in two cell lines: TE-1 and Eca-109, which were selected for the next experiments. To investigate the role of HOXA10 in the proliferation of EC cells, HOXA10 was overexpressed and knocked down. As shown in Figure 2a and b, both mRNA and protein levels of *HOXA10* were enhanced in Eca-109 and TE-1 cells with HOXA10 overexpression, whereas siHOXA10 had the opposite effect on HOXA10 expression (Figure A1). As shown in Figure 2c, it was revealed that overexpression of HOXA10 promoted the Eca-109 and TE-1 cell viability, whereas HOXA10 knockdown suppressed cell viability (Figure 2c). Interestingly, overexpression of HOXA10 increased the colony number and siHOXA10 decreased the colony number (Figure 2d). These findings indicated that overexpression of HOXA10 promoted EC cells’ proliferation.

### 3.3 Overexpression of HOXA10 suppressed cell apoptosis in EC cells

**Figure 3:**
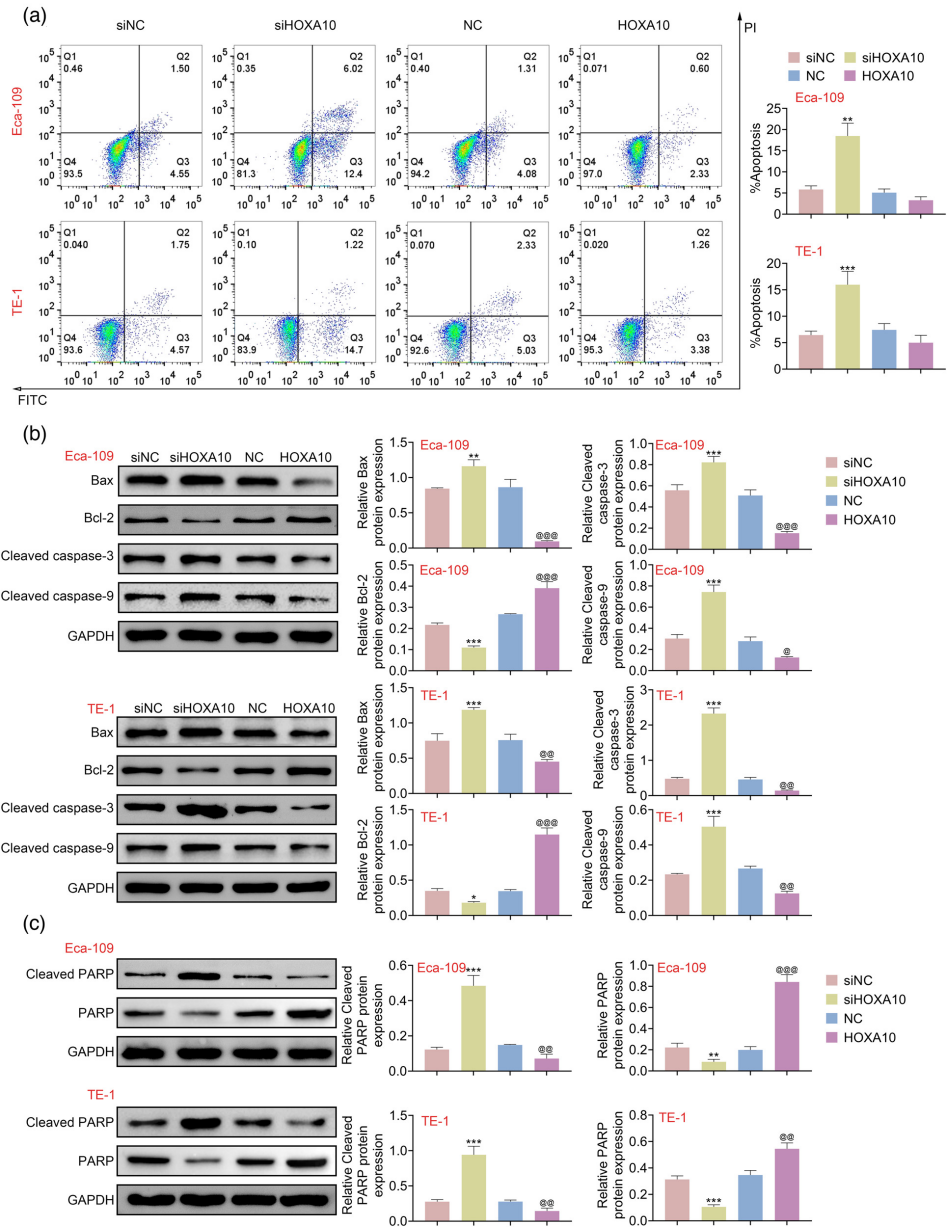
Overexpression of HOXA10 suppressed cell apoptosis in EC cells. (a) The siHOXA10 increased apoptosis rate of Eca-109 and TE-1 cells and overexpression of HOXA10 increased apoptosis rate. Annexin V-FITC assay was used to measure cell apoptosis. (b) The apoptosis-related proteins containing Bax, Bcl-2, cleaved caspase-3, and cleaved caspase-9 were determined by western blot Eca-109 and TE-1 cells. (c) The cleaved PARP and PARP protein levels were assessed by western blot. Each experiment repeated three times. ^*^ *p*  <  0.05, ^**^ *p*  <  0.01, ^***^ *p*  < 0 .001 vs NC. ^@^ *p* < 0.05, ^@@^ *p* < 0.01, ^@@@^ *p* < 0.001 vs siNC.

To claim the function of HOXA10 on cell apoptosis in EC cells, the apoptosis was evaluated by Annexin V-FITC assay. As illustrated in Figure 3a, overexpression of HOXA10 showed a significant decrease in apoptosis rate; nevertheless, knockdown of HOXA10 showed a markedly increase in apoptosis rate in TE-1 and Eca-109 cells (Figure A1). Consistently, western blot showed that cleaved caspase-9, Bax, and cleaved caspase-3 expressions were substantially enhanced by siHOXA10, while Bcl-2 expression was markedly reduced in TE-1 and Eca-109 cells, which have opposite effects in TE-1 and Eca-109 cells transfected with oe-HOXA10 (Figure 3b). More importantly, the cleaved PARP protein was accumulated in siHOXA10 cells, while PARP was reduced in siHOXA10 cells. Nevertheless, overexpressed HOXA10 decreased cleaved PARP expression and increased PARP expression (Figure 3c). These findings indicated that overexpression of HOXA10 suppressed cell apoptosis in EC cells.

### 3.4 HOXA10 activated p38/ERK signaling pathway in EC cells

**Figure 4:**
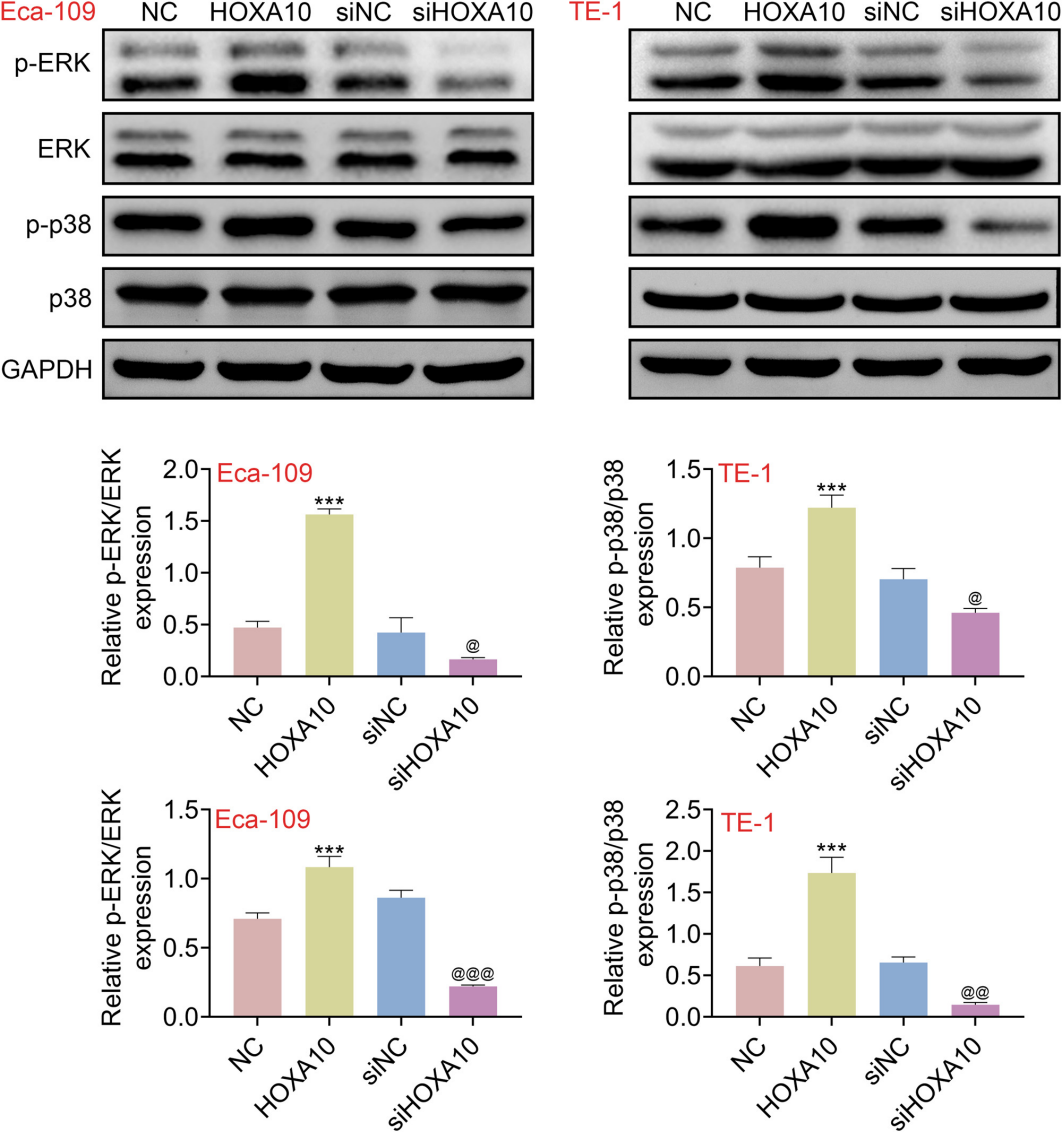
HOXA10 activated p38/ERK signaling pathway in EC cells. After transfected with oe-HOXA10 or siHOXA10, the p-ERK, ERK, p-P38, and P38 expressions were detected by western blot in Eca-109 and TE-1 cells. Each experiment repeated three times. ^***^ *p*  < 0 .001 vs NC. ^@^ *p* < 0.05, ^@@^ *p* < 0.01, ^@@@^ *p* < 0.001 vs siNC.

To claim the mechanism underlying the pro-proliferative and anti-apoptotic function of HOXA10 in EC cells, western blot assays were conducted. As shown in Figure 4, the ratio of p-ERK/ERK was elevated by the overexpression of HOXA10, which was reduced by siHOXA10 in TE-1 and Eca-109 cells. More importantly, the ratio of p-p38/p38 was increased by the overexpression of HOXA10, which was decreased siHOXA10 by in TE-1 and Eca-109 cells. In conclusion, these results concluded that HOXA10 activated p38/ERK signaling pathway in EC cells.

## 4 Discussion

In this study, HOXA10 was observed to highly express in EC tissues and cells. Interestingly, overexpression of HOXA10 enhanced proliferation and suppressed cell apoptosis in EC cells. More importantly, overexpression of HOXA10 activated p38/ERK signaling pathway in EC cells. These findings concluded that HOXA10 aggravated EC progression via activating p38/ERK signaling pathway, providing a potential therapeutic target for EC.

Accumulating evidence has reported that diverse kinds of molecules participate in the regulation of EC progression. Circular RNA LPAR3 sponges microRNA-198 to facilitate EC proliferation and metastasis [14]. Moreover, PAQR3 suppresses the EC cells’ proliferation and tumorigenesis [15]. Wang et al. have demonstrated that miR-30e downregulation increases EC cell proliferation, invasion, and tumor growth through targeting RPS6KB1 [16]. Given that the poor prognosis of EC patients, it is essential to find out more small molecules, which can be potential therapeutic targets or biomarkers for EC. In this study, our results confirmed that HOXA10 expression was enhanced in EC cells, which was consistent with the expression trend in TCGA data. Accumulating evidence has reported that HOXA10 serves as a carcinogenesis in the progression of various human cancers. It has been reported that HOXA10 deteriorates gastric cancer through inducing Bcl-2 expression and activating JAK1/STAT3 signaling pathway [6,17]. HOXA10 suppression inhibited proliferation and enhanced apoptosis of head and neck squamous cell carcinoma cells [18]. Similarly, we demonstrated that overexpression of HOXA10 promoted EC cells proliferation and inhibited cell apoptosis, which might be the first time clarifying the potential role of HOXA10 in EC. However, the mechanism of HOXA10 in EC progression is still unknown.

Unlimited cell proliferation and loss of apoptosis are vital biological characteristics of tumors [19]. The activation of ERK signaling pathway has pro-proliferative and anti-apoptotic effect [20]. Inhibition of the expression of this pathway can suppress the tumor cells proliferation and loss of apoptosis, thereby promoting the differentiation of tumor cells [21]. ERK signaling pathway has been identified as a potential pro-tumorigenic promoter in EC progression, which has been hyperactivated in EC [22]. NETO2 promotes EC progression by inducing proliferation and metastasis via increased the phosphorylation of ERK [23]. Macrophage stimulating 1 promotes the anti-tumor effects in EC via inactivating the ERK signaling pathway [24]. Similarly, we demonstrated that high expression of HOXA10 was associated with the activating of ERK signaling pathway, which obviously explained the pro-proliferative and anti-apoptotic effect of HOXA10 in EC.

The p38 signaling pathway has links with ERK signaling pathway, and these pathways belong to mitogen-activated protein kinase (MAPK) signaling pathway. Initially, p38 is activated by proinflammatory cytokines, stress stimuli, and growth factors. Like the certain finding of pro-tumorigenic activity of ERK in EC, EC progression dependency on p38 signaling pathway hyperactivation is similar to the established anti-tumorigenic of ERK signaling pathway. Coptisine inhibited EC cells’ proliferation via the inhibition of p38/ERK signaling pathway [25]. Consistently, in this study, we confirmed that high expression of HOXA10 was related to the activating of p38 signaling pathway, indicating that the pro-proliferative and anti-apoptotic effect of HOXA10 in EC depended on the activation of p38 signaling pathway. Besides, the change of p-ERK is more obvious than that of p38 after overexpression of HOXA10 in the ECA109 cells, which was opposite in the TE-1 cell, maybe owing to the different cell properties of different cell lines. This might be a novel sight for investigating the tumorigenesis of EC.

Yet, limitations exist in the study that the clinical data in EC patients need to be enlarged in future studies. Due to the limited funding, there were no animal experiments to support our conclusion. The results would be more reliable if the limitations can be made up. Moreover, given the potential role of HOXA10, the regulation of HOXA10 expression involved in carcinogenesis and cancer inhibition, which should be explored in further investigation.

In summary, our results demonstrated that HOXA10 was highly expressed in EC tissues and cells, more importantly, overexpression of HOXA10 promoted EC cells’ proliferation and suppressed apoptosis. Therefore, HOXA10 may act as a therapeutic target for EC.
